# Active surveillance of acute paediatric hospitalisations demonstrates the impact of vaccination programmes and informs vaccine policy in Canada and Australia

**DOI:** 10.2807/1560-7917.ES.2020.25.25.1900562

**Published:** 2020-06-25

**Authors:** Karina A Top, Kristine Macartney, Julie A Bettinger, Ben Tan, Christopher C Blyth, Helen S Marshall, Wendy Vaudry, Scott A Halperin, Peter McIntyre, on behalf of the IMPACT and PAEDS investigators

**Affiliations:** 1 Department of Pediatrics, Dalhousie University, Halifax, Canada; 2 Canadian Center for Vaccinology, IWK Health Centre, Halifax, Canada; 3 These authors contributed equally; 4 National Centre for Immunisation Research and Surveillance and The Children’s Hospital Westmead, Sydney, Australia; 5 Faculty of Medicine and Health, The University of Sydney, Sydney, Australia; 6 University of British Columbia and Vaccine Evaluation Center, British Columbia Children’s Hospital, Vancouver, Canada; 7 University of Saskatchewan, Royal University Hospital, Saskatoon, Canada; 8 Telethon Kids Institute and School of Medicine, University of Western Australia and Perth Children’s Hospital, Perth, Australia; 9 Robinson Research Institute and Adelaide Medical School, The University of Adelaide and VIRTU Women’s and Children’s Health Network, Adelaide, Australia; 10 University of Alberta, Stollery Children’s Hospital, Edmonton, Canada; 11 The IMPACT and PAEDS investigators are acknowledged at the end of this article

**Keywords:** COVID-19, emerging infectious diseases, immunisation, SARS-CoV-2, surveillance, vaccination, vaccine-preventable disease, pandemic influenza, syndromic conditions, paediatric hospital surveillance

## Abstract

Sentinel surveillance of acute hospitalisations in response to infectious disease emergencies such as the 2009 influenza A(H1N1)pdm09 pandemic is well described, but recognition of its potential to supplement routine public health surveillance and provide scalability for emergency responses has been limited. We summarise the achievements of two national paediatric hospital surveillance networks relevant to vaccine programmes and emerging infectious diseases in Canada (Canadian Immunization Monitoring Program Active; IMPACT from 1991) and Australia (Paediatric Active Enhanced Disease Surveillance; PAEDS from 2007) and discuss opportunities and challenges in applying their model to other contexts. Both networks were established to enhance capacity to measure vaccine preventable disease burden, vaccine programme impact, and safety, with their scope occasionally being increased with emerging infectious diseases’ surveillance. Their active surveillance has increased data accuracy and utility for syndromic conditions (e.g. encephalitis), pathogen-specific diseases (e.g. pertussis, rotavirus, influenza), and adverse events following immunisation (e.g. febrile seizure), enabled correlation of biological specimens with clinical context and supported responses to emerging infections (e.g. pandemic influenza, parechovirus, COVID-19). The demonstrated long-term value of continuous, rather than incident-related, operation of these networks in strengthening routine surveillance, bridging research gaps, and providing scalable public health response, supports their applicability to other countries.

## Background

The essential role of hospital-based sentinel surveillance in identifying emerging infections and measuring the incidence of severe disease was highlighted by Thomson and Nicoll in a 2010 editorial in *Eurosurveillance* referring to surveillance activities in Europe during the 2009 influenza A(H1N1)pdm09 pandemic [1]. They argued that given demands on clinicians in disease outbreaks, sentinel hospital-based surveillance needed to be ongoing, allowing it to be augmented, rather than established de novo, in times of crisis [1]. They identified a range of potential roles for sentinel hospital networks in Europe, primarily collection of clinical data, linked to laboratory and epidemiological data, supporting rapid, evidence-based outbreak responses. Beyond outbreak response, hospital networks have been established in low- middle- and high-income countries to provide quality data for immunisation programmes, infection control, and seasonal influenza [2-8].

Most hospital-based surveillance networks are focused on specific diseases or syndromic targets. This article analyses the contributions of two active, paediatric hospital-based sentinel networks that, since their inception, have played wide-ranging roles in public health surveillance: the Canadian Immunization Monitoring Program, Active (IMPACT) established in 1991, and the Australian Paediatric Active Enhanced Disease Surveillance (PAEDS) network established in 2007. These networks, which contribute to surveillance of syndromic conditions, vaccine-preventable diseases (VPDs), vaccine safety monitoring, and emerging infections, are unique in the range of conditions under surveillance and potential for scalability in response to public health emergencies. We present evidence on their feasibility and value to public health surveillance, and discuss opportunities for similar platforms to enhance public health surveillance in low-, middle-, and high-income countries.

## History and contributions

Both IMPACT and PAEDS arose from identified surveillance gaps in child health outcomes related to vaccine safety and VPDs. Addressing these required the establishment of national surveillance programmes due to the relatively small numbers of both paediatric patients and tertiary care centres in Canada and Australia.

### Canada

The need for a hospital-based active surveillance system to reliably detect serious adverse events following immunisation (AEFIs) was recognised following detection of an increase in aseptic meningitis associated with the Urabe mumps vaccine strain in 1986–1988 by virologists at several Canadian children’s hospitals [9]. Investigation of this signal, not identified by the passive system, led to replacement of the Urabe vaccine with a safer vaccine. IMPACT began in 1991 as a collaboration between Health Canada and the Canadian Paediatric Society (CPS) at five paediatric tertiary care centres in five provinces. The first surveillance targets included neurological admissions (e.g. acute flaccid paralysis (AFP), encephalopathy, seizure) and several VPDs (e.g. pertussis) (Table 1 and Supplemental Content 1) [9]. IMPACT expanded to 12 centres in eight provinces by 1999, capturing approximately 90% of paediatric tertiary care beds in Canada [9].

**Table 1:** Canadian IMPACT and Australian PAEDS surveillance targets and years of surveillance

Target	Years of surveillance	Integration with other surveillance systems	Funding
**AEFIs**			
IMPACT (established 1991)			
AFP including Guillain–Barré syndrome	1991–present	Reports to Canadian Paediatric Surveillance Program (AFP), Canadian Adverse Event Following Immunization Surveillance System and provincial/territorial public health	PHAC
Encephalopathy/encephalitis/myelitis	1991–present		
Bell's palsy	1991–present		
Seizure	1991–present		
Hypotonic hyporesponsive episode	1991–2012		
Thrombocytopaenia	1991–present		
Injection site reactions (cellulitis, abscess)	1991–present		
Complications of vaccination	2013–present		
Intussusception	2009–present		
Varicella vaccine reactivation illness	2013–present		
Other reportable AEFIs identified while searching for the above (e.g. anaphylaxis)	1991–present		
PAEDS (established 2007)			
AFP including Guillain–Barré syndrome	2007–present	Communicable Diseases Network of Australia’s polio expert panel AEFI captured as part of AFP surveillance (see below)	Commonwealth state/territory governments
Severe acute neurological events	2013–present	Commonwealth government (Office of Health Protection) and state/territory governments	
Intussusception	2007–present		
Seizures	Infant seizures: 2007–2008 Febrile seizures: 2013–2014		NHMRC
**VACCINE-PREVENTABLE DISEASES**			
IMPACT			
Pertussis	1991–present^a^	Complements CNDSS	PHAC
Invasive *Haemophilus influenzae* disease	Type b: 1991–present All types: 2007–present		
Congenital rubella syndrome	1991–1998		
Varicella zoster	2000–present		
Influenza	2004–present	Reports to national FluWATCH system	
Invasive pneumococcal disease	1991–present	Complements CNDSS	Industry funded 1999–2004; currently funded by PHAC
Invasive meningococcal disease	2002–present	Complements CNDSS	Industry funded
Rotavirus	2005–2019	No existing public health surveillance	Industry funded
PAEDS			
Varicella and zoster	2007–present	No nationally consistent surveillance	Commonwealth and state governments
Influenza	2009 2014–present	Reports via FluCAN	Commonwealth government NHMRC
Pertussis Invasive meningococcal disease	2012–present 2015–present	Complements national dataset National Neisseria Network	NHMRC State governments
COVID-19 and PIMS-TS	2020–present	Rapidly activated; COVID-19 reports via FluCAN	Commonwealth and state governments
**OTHER**			
IMPACT			
Respiratory syncytial virus	2017–2020	No existing public health surveillance	PHAC
PAEDS			
Acute encephalitis^b^	2013–present	Commonwealth government (Office of Health Protection) state/territory governments	Investigator funded^b^ Commonwealth and state/territory governments NHMRC
Respiratory syncytial virus^b^	Pilot study 1 site, 2018		
Invasive group A streptococcal disease^b^	Pilot study multiple sites, 2018		
Kawasaki disease	2018–present		

AEFI: adverse events following immunisation; AFP: acute flaccid paralysis; CNDSS: Canadian Notifiable Disease Surveillance System; COVID-19: coronavirus disease; CPSP: Canadian Paediatric Surveillance Program; FluCAN: InFLUenza Complications Alert Network; IMPACT: Canadian Immunization Monitoring Program, Active; NHMRC: National Health and Medical Research Council; PAEDS: Paediatric Active Enhanced Disease Surveillance; PHAC: Public Health Agency of Canada; PIMS-TS, pediatric inflammatory multisystem syndrome temporally associated with SARS-CoV-2; SARS-CoV-2: severe acute respiratory syndrome coronavirus 2.

^a^ From 1991 to 1998 only children < 2 years of age were included.

^b^ Investigator funding includes institutional trainee scholarships and fellowships.

For 29 years, IMPACT has collected epidemiological data for AEFIs and diseases that are current or future targets for vaccine prevention, demonstrating the effectiveness of new immunisation programmes, including, meningococcal conjugate, pneumococcal conjugate and varicella vaccines (Table 2) [10-12].

**Table 2:** Major accomplishments of IMPACT since its inception, Canada, 1991–2019

Surveillance target	Major findings	Impact	Selected references^a^
**Adverse events following immunisation**			
Infectious complications of vaccination	150-fold higher than expected incidence of disseminated BCG disease among Indigenous children	Routine use of BCG limited to communities with ongoing active TB disease, with negative HIV screening and no risk factors for PID	Deeks, 2005 [30]; Scheifele, 1998 [31]
HHE	67% decrease in HHE after aP vs wP	First evidence of improved safety profile of aP over wP	Le Saux, 2003 [32]
Seizure	79% decrease in seizure after aP vs wP	First evidence of improved safety profile of aP over wP	Le Saux, 2003 [32]
Thrombocytopaenia	Two of 107 children admitted with post-immunisation thrombocytopaenia had severe bleeding and 93% recovered within 3 months	Largest cohort of post-immunisation thrombocytopaenia	Jadavji, 2003 [33]; Sauvé, 2010 [34]
**Vaccine-preventable diseases**			
*Haemophilus influenzae*	95–99% reduction in invasive Hib cases following introduction of infant Hib immunisation programmes; emergence of Hia in Indigenous populations; children with cancer > 5 years of age are susceptible to invasive Hib	Demonstrated effectiveness of Hib vaccination programmes and provided new data to support development of Hia vaccine	Scheifele, 1996; McConnell, 2007; Tan, 2016; McNair, 2018 [35-38];
IPD	48% decrease in IPD from pre-PCV to PCV13 era; IPD due to PCV13 serotypes decreased from 89% to 34% of cases	Demonstrated effectiveness of PCV vaccination programmes in Canada and changing epidemiology of IPD	Bettinger, 2010 [10]; Bettinger, 2016 [39]
Invasive meningococcal disease	69% reduction in meningococcal serogroup C disease following implementation of meningococcal C conjugate vaccine programmes; shift to serogroup B as predominant cause of IMD	Demonstrated effectiveness of infant and adolescent meningococcal C vaccination programmes and estimated benefit of introducing meningococcal B vaccination	Bettinger, 2013 [40]; Sadaranagani, 2014 [11];
Pertussis	Documented changing epidemiology of pertussis from wP to aP eras; in aP era, 76% of hospitalised cases and all 21 deaths were infants 0–3 months of age	Demonstrated ongoing burden of pertussis in young infants suggesting potential benefit of Tdap vaccination during pregnancy	Halperin, 1999; Bettinger, 2007; Abu Raya, 2020 [41-43]
Rotavirus	83% reduction in rotavirus hospitalisations at centres with infant immunisation programmes	Contributed data to support implementation of rotavirus immunisation programmes in Canada and demonstrated the benefits of those programmes	Le Saux, 2010 [44]; Le Saux, 2016 [45]
Varicella	85% reduction in varicella-related hospitalisations following introduction of two-dose varicella immunisation programmes	Early evidence of the effectiveness of single-dose varicella immunisation programmes and added benefit of second dose in reducing hospitalisation	Law, 2000 [46]; Tan, 2012 [12]; Tan, 2018 [47]
Influenza	Reported on relative severity of influenza B vs A in children and high risk of influenza-related complications in children with neurodevelopmental conditions	Provided evidence to support use of quadrivalent influenza vaccines in children and addition of neurological and neurodevelopmental conditions to high-risk conditions for influenza vaccination	Tran, 2012; Burton, 2014; Tran, 2016 [48-50]

aP: acellular pertussis vaccine; BCG: Bacillus Calmette-Guérin vaccine; HHE: hypotonic hyporesponsive episode; Hia: *Haemophilus influenzae* type a; HIV: human immunodeficiency virus; IMD: invasive meningococcal disease; IMPACT: Canadian Immunization Monitoring Program, Active; IPD: invasive pneumococcal disease; PCV: pneumococcal conjugate vaccine; PID: primary immunodeficiency; TB: tuberculosis; Tdap: tetanus-diphtheria-acellular pertussis vaccine; wP: whole cell pertussis vaccine.

^a^ For additional publications, see also: https://www.cps.ca/en/impact.

### Australia

The PAEDS system was established in 2007 to support Australian compliance with World Health Organization (WHO) AFP surveillance standards as part of polio eradication efforts, and to conduct surveillance for varicella hospitalisations following vaccine introduction and two AEFIs potentially associated with varicella and rotavirus vaccination programmes (seizures and intussusception) (Table 1 and Supplemental Content 1) [13]. PAEDS was funded by the Australian Government as a pilot project in four paediatric hospitals in four states. PAEDS subsequently expanded to seven hospitals in six states and territories, covering around 80% of tertiary paediatric beds. The scope of PAEDS was enlarged over time to provide key evidence regarding vaccine effectiveness, safety, and the impact of new vaccination programmes and to increase the population under surveillance (Table 3 and Supplemental Content 1).

**Table 3:** Major accomplishments of PAEDS since its inception, Australia, 2007–2019

Surveillance target	Major findings	Impact	Selected references^a^
**AEFIs**			
IS	First to publish low but increased vaccine risk of IS following rotavirus vaccine with new second generation vaccines (RotaTeq and Rotarix); further confirmed risk (vaccine attributable risk of 6/100,000) and risk–benefit of vaccine programmes, and demonstrated that vaccine-associated IS is not more severe than non-vaccine associated IS	Provided globally relevant safety data on new vaccines, cited by WHO and multiple other peak immunisation advisory committees; informed risk–benefit considerations regarding ongoing rotavirus vaccination programmes	Buttery, 2011; Carlin, 2013; Quinn, 2014 [51-53]
FS following immunisation	Demonstrated absence of risk of FS following MMRV vaccine when used as second dose of measles-containing vaccine in children aged 12–24 months, and known risk of FS post-MMR dose 1 vaccine, with no risk post monovalent-varicella vaccine; clinical severity and developmental outcomes associated with vaccine-proximate seizures in children not different to children with non-vaccine proximate seizures	Provided important safety outcome monitoring relevant to NIP new vaccine introduction (MMRV vaccine in 2013); research into vaccine proximate seizures provided new insights and reassurance for public and immunisation providers	Deng, 2019; Macartney, 2015; Macartney, 2017 [54-56]
SANE following immunisation	Includes acute disseminated encephalomyelitis, AFP, GBS and transverse myelitis; monitoring of case numbers where receipt of vaccination occurred in previous 6 weeks for reporting as potentially severe AEFI; GBS cases post-influenza A(H1N1)pdm09 vaccine contributed to a multinational study of influenza A(H1N1)pdm09 vaccine safety	Provided reassurance of influenza (pandemic and seasonal) and other vaccine safety with regard to SANEs; contributed to multi-country global analysis of GBS following pandemic influenza vaccine	Dodd, 2013; McRae, 2019 [57,58]
**Vaccine-preventable diseases**			
2009 influenza A(H1N1)pdm09 pandemic	Demonstrated impact of 2009 influenza A(H1N1)pdm09 pandemic on children, extensively documenting hospitalised disease fraction	Key data source to measure impact and outcomes from 2009 influenza A(H1N1)pdm09 pandemic in children	Khandaker, 2011 [59]; Khandaker, 2012 [60]; Khandaker, 2014 [21]
Seasonal influenza	Data on vaccination, including in pregnancy for infants aged < 6 months, collected to calculate vaccine effectiveness; contributed samples for national genotyping; demonstrated paediatric disease burden and vaccine effectiveness over multiple seasons, as well as providing novel data on serious complications, such as influenza-associated encephalopathy	In 2017, detailed data on extensive and severe disease from influenza in children; informed newly funded paediatric influenza programmes in 6 states and territories	Blyth, 2016 [61]; Blyth, 2019 [2]; Cheng, 2017a; Cheng, 2017b; Li-Kim Moy, 2017 [62-64]
Invasive meningococcal disease	Additional detailed data to complement National Notifiable Disease Surveillance System, as well as long-term follow-up of outcomes and complications.	Evidence for severe outcomes and healthcare- associated costs, assisting in informing policymakers regarding new programmes	McRae, 2019 [58]
AFP (poliovirus)	Report ca 80% of all AFP cases as part of Australian acute flaccid surveillance and enteric virus surveillance programmes; post discharge follow-up and collection of faecal samples for detailed testing	Enables Australia to fulfil WHO requirements for AFP surveillance; assisted in documentation of new emerging pathogens, e.g. EV71 and parechovirus	Paterson, 2013 [13]; McRae, 2019 [58]
Pertussis	Demonstrated severity of early infant disease, and decline in hospitalised pertussis following introduction of maternal vaccination	Evidence for impact of maternal pertussis vaccination in Australia	Quinn, 2018 [65]
Varicella	Documented decline in hospitalised varicella following one-dose vaccine programme introduction; provided longitudinal data on varicella genotyping over 10 years; documented rare but complex cases of vaccine virus associated disease	Key evidence of vaccine programme impact and of moderate vaccine effectiveness of one-dose schedule under NIP; association of European clade with severity of hospitalised cases	Marshall, 2013 [66]; Marshall, 2019 [67]; Quinn, 2019 [53]
**Others**			
Acute encephalitis	Provided detailed analysis on aetiology, epidemiology, outcomes and healthcare needs of acute childhood encephalitis, particularly relevant to communicable disease control, such as influenza, EV71, parechovirus, mycoplasma and vector borne diseases	Work supported development of national clinical guideline for investigation and management, provided early detection of EV71 and parechovirus disease outbreaks, and provided data for new influenza vaccine programme introduction in children	Britton, 2016a [68]; Britton, 2016b [17]; Britton, 2017 [69]
iGAS	Pilot study demonstrated clinical severity and epidemiology of children hospitalised with iGAS	Informed public health guidance and consideration of iGAS to be a nationally notifiable condition	Thielemans, 2020 [70]
RSV	Pilot study demonstrated clinical severity of disease in hospitalised infants with RSV	Pilot demonstrated feasibility of providing detailed baseline (pre-vaccine introduction) data on disease burden to inform economic evaluation and contributed to WHO Global RSV Surveillance Pilot study	Hirve et al, 2019 [71]

AEFI: adverse event following immunisation; AFP: acute flaccid paralysis; EV71: enterovirus 71; FluCAN: InFLUenza Complications Alert Network; FS: febrile seizure; GBS: Guillain–Barré syndrome; iGAS: invasive Group A streptococcal disease; IS: intussusception; MMRV: measles-mumps-rubella-varicella vaccine; NIP: national immunisation programme; PAEDS: Paediatric Active Enhanced Disease Surveillance; RSV: respiratory syncytial virus; SANE: severe acute neurological events; WHO: World Health Organization.

^a^ For additional publications, see also: www.paeds.edu.au.

## Network organisation and procedures

Both IMPACT and PAEDS utilise the contribution of trained surveillance nurses at each hospital, supervised by volunteer paediatric clinicians who act as site investigators. Nurses screen hospital and emergency department admission lists for conditions under surveillance, review medical records, retrieve immunisation records, and report cases electronically on standardised case report forms to the national coordinating centre (Figure and Table 1).

**Figure:**
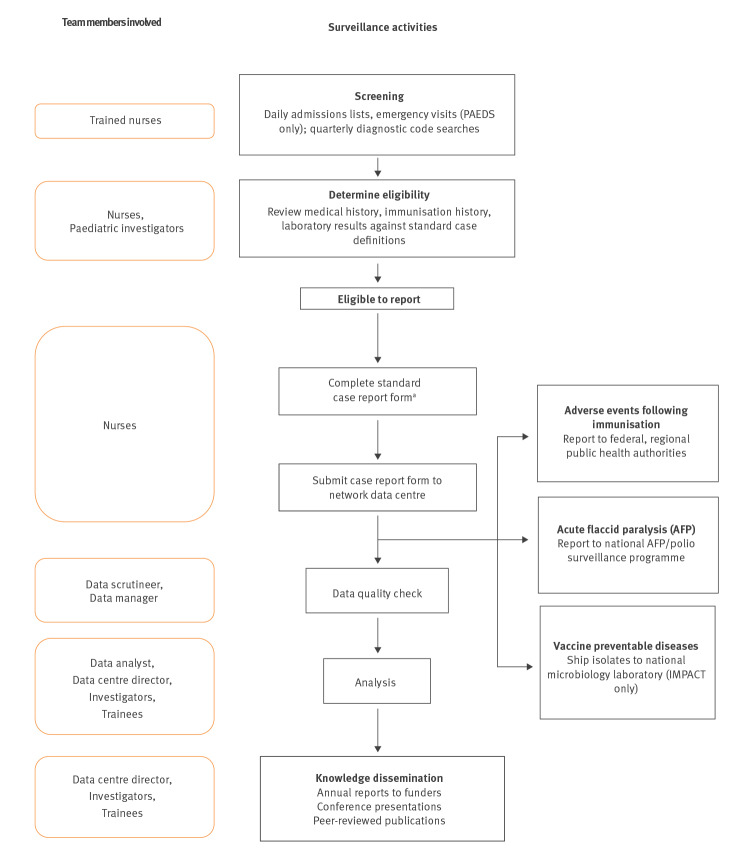
Surveillance approaches of IMPACT and Australian PAEDS programmes

IMPACT: Immunization Monitoring Program, Active; PAEDS: Paediatric Active Enhanced Disease Surveillance.

^a^ PAEDS network requires consent for select conditions and/or for enhanced data collection.

The national coordinating centre submits data to national public health authorities at least quarterly for incorporation into national datasets. In Canada and Australia, centres report AEFIs directly to regional and national public health authorities.

Annual in-person meetings and standardised training have been important to maintain group cohesion and national consistency, while opportunities for data analysis, peer-reviewed publications, and improved policy and practice sustain investigator engagement.

### Ethical statement

IMPACT and PAEDS surveillance is conducted and reported in line with the Declaration of Helsinki, as revised in 2013. Ethics and/or hospital approvals are in place at participating institutions (Supplemental Content 2).

### Funding and resources

IMPACT is supported primarily by federal funding and managed by a non-profit organisation (CPS). This unique arrangement has allowed alternate sources of funding from provincial governments and industry to augment federally funded activities and support additional surveillance targets (e.g. rotavirus), providing stability for the network, while ensuring investigators retain independence in data collection, analysis, and publication.

PAEDS is supported by federal, state and territory government funding, and provides a platform for researchers to use the PAEDS infrastructure on a cost-recovery basis for other serious childhood conditions, such as Kawasaki disease. PAEDS has not received pharmaceutical industry funding.

Network funding, together with funding garnered to add new conditions, provides part-time support for one nurse per site, a national nurse coordinator, and data centre staff. Site investigators provide in-kind support.

## Hospital-based surveillance complements public health surveillance

### Canada

Public health surveillance of select VPDs is mandated by provincial and territorial governments with voluntary reporting to the Canadian Notifiable Disease Surveillance System but captures only disease onset date, sex and age. IMPACT captures additional variables for hospitalised cases including co-morbid conditions, concurrent and past infections, immunisation history, need for intensive care, and outcome at discharge. This information allows in-depth characterisation of disease burden and risk groups, as well as estimation of vaccine effectiveness, and informs cost-effectiveness analyses. Biological specimens are collected for select VPDs (e.g. *Streptococcus pneumoniae, Neisseria meningitidis,* rotavirus) enabling strain characterisation and monitoring for strain replacement (Table 2) [10].

IMPACT provides the only information on paediatric hospital admissions for influenza in Canada. IMPACT data are incorporated into ‘FluWatch’, Canada's national influenza and influenza-like illnesses surveillance system [14]. IMPACT’s weekly reporting during the influenza season allows public health to assess influenza transmission and severity by person, place and time, as well as the impact and burden of influenza epidemics in real time.

The Canadian AEFI Surveillance System (CAEFISS), the national post-market vaccine safety monitoring system, relies primarily on spontaneous reporting of AEFIs to public health [15]. IMPACT contributes > 50% of serious AEFIs and 70–90% of neurological AEFIs reported to CAEFISS [15]. In 1998, IMPACT identified an increase in disseminated Bacillus Calmette–Guérin (BCG) disease in Indigenous children with undiagnosed primary immunodeficiency, prompting changes to BCG vaccination recommendations in Canada [9].

### Australia

Due to the existence of robust laboratory-based VPD surveillance through the National Notifiable Diseases Surveillance Scheme, which also captures biological specimens for select VPDs (such as those described for Canada) [16], the PAEDS network has focused on conditions where there is syndromic diagnosis (e.g. AFP, encephalitis), limited sensitivity or utilisation of laboratory tests (e.g. varicella), or where gaps in capture of immunisation status and clinical severity exist (e.g. paediatric influenza).

After 2007, PAEDS emerged as the reporting source for ca 80% of AFP cases to the Communicable Diseases Network of Australia’s polio expert panel [13], resulting in Australia consistently exceeding the WHO reporting target. Enhanced studies of encephalitis via PAEDS facilitated evaluation of emerging viral infections, including enterovirus 71 and parechovirus (Table 3) [17].

Australia monitors influenza activity through a variety of complementary surveillance systems [18]. Sentinel hospital surveillance for influenza is conducted in collaboration with the InFLUenza Complications Alert Network (FluCAN) [18], which captures data from 22 sites across Australia, including seven PAEDS sites, enabling real-time tracking of a representative number of children. Paediatric influenza surveillance, including characterisation of over 1,300 paediatric hospitalisations during the 2017 influenza season, provided evidence to prompt state and territory funding of influenza vaccines for children aged 6–59 months from 2018, and inclusion on the National Immunisation Program from 2020 [2,19].

### Rapid response capacity

Both networks demonstrated capacity to respond to outbreaks of emerging diseases during the 2009 influenza A(H1N1)pdm09 pandemic. IMPACT scaled up its influenza activities to continue throughout the summer (June–August) and provided one of the earliest reports on the paediatric burden of influenza A(H1N1)pdm09 disease in the Northern Hemisphere during the first pandemic wave [20]. PAEDS developed questionnaires and protocols for identifying hospitalised cases of influenza rapidly, following pilot work in individual hospitals [21]. During the coronavirus disease (COVID-19) pandemic, PAEDS has been capturing data on laboratory-confirmed severe acute respiratory syndrome coronavirus 2 (SARS-CoV2) infections leading to hospitalisation or Emergency Department visit since March 2020. Surveillance for Paediatric Inflammatory Multisystem Syndrome Temporally associated with SARS-CoV-2 (PIMS-TS; and also known as Multisystem Inflammatory Syndrome in Children (MIS-C) in the USA), a newly described inflammatory syndrome occurring during or after SARS-CoV-2 infection in children, commenced in May 2020 [22,23].

## Representativeness and ethics

IMPACT and PAEDS networks are based in paediatric referral centres and therefore do not cover the whole population. Calculation of disease incidence has been limited to severe diseases, such as invasive meningococcal disease (IMPACT) [11] or encephalitis (PAEDS), where either most paediatric cases are admitted or transferred to an IMPACT or PAEDS centre, or the incidence is low enough to also capture cases admitted to regional or community hospitals.

To ensure complete case capture, IMPACT operates without obtaining informed consent or enrolling individual participants. This requires the data collected to be available in a hospital chart or immunisation record. Patients or caregivers are not able to add or clarify missing data.

PAEDS originally commenced surveillance requiring informed consent to allow patients to enrol for data collection. However, this resulted in non-inclusion of patients whose parents had limited spoken English or health literacy. PAEDS now operates under a national ethical framework that allows capture of a minimal de-identified dataset for all cases. Parents or caregivers can ‘opt out’ of their data being used and consent is still obtained to gain additional information or to opt into additional studies via parent/caregiver interview.

Another challenge faced by both systems relates to variations in capacity in the event of severe disease epidemics. During the record breaking 2017 influenza season in Australia [2], a fivefold increase in hospitalisations (cf.d with previous years) diverted nurse time away from prompt recording of other surveillance conditions.

## Opportunities for active hospital-based surveillance

Hospital-based surveillance systems in high-, middle- and low-income countries, such as the Influenza Monitoring of Vaccine Effectiveness Network (I-MOVE), Healthcare-associated Infections Surveillance Network (HAI-Net) in Europe, Global Rotavirus and Invasive Bacterial Vaccine Preventable Diseases Surveillance Networks (IB-VPD), and AEFI surveillance network in the Americas have generally focused on a specific disease or syndromic target [3-7,24,25]. However, collectively they represent surveillance activities similar to IMPACT or PAEDS.

Population registries and linked databases have also been used to evaluate vaccine safety and effectiveness [26,27]. However, they are limited to high-income countries, case capture may be incomplete for certain conditions (e.g. varicella), and capacity for rapid response, detailed clinical data collection and linkage to biological specimens varies [28].

IMPACT and PAEDS have demonstrated that the same platform and similar surveillance methodologies can be applied to study a broad range of diseases and syndromes of public health importance. Conditions under surveillance can be added in response to new vaccines, vaccine safety concerns and emerging diseases, while others can be discontinued or modified. This provides efficiencies with respect to staffing time, as well as flexibility and responsiveness in the event of disease outbreaks. When emerging diseases or other conditions of concern arise, established networks like I-MOVE, IB-VPD may be well placed to rapidly expand their surveillance targets. Sharing of standard surveillance protocols may also help low- and middle-income countries expand their surveillance capacity [25]. Exploration of this concept may warrant incorporation of hospital-based surveillance networks into emerging infectious disease and AEFI surveillance plans. Capacity to activate surveillance platforms to rapidly respond to communicable disease emergencies, particularly those threatening global health security, such as the COVID-19 pandemic, is recognised as essential [29].

## Conclusions

IMPACT and PAEDS have been implemented successfully to address gaps in, and add value to, public health surveillance in two countries with different needs and health systems. The adaptability of both networks to changing public health priorities in their respective countries has been critical to their success. Active hospital-based sentinel surveillance systems can leverage efficiencies gained by monitoring for more than one condition to play multiple roles in informing public health policy and responding to public health emergencies. Existing surveillance systems should consider their potential to expand conditions under surveillance, particularly as the need to evaluate health interventions and monitor for emerging infectious diseases, such as COVID-19 grows.

## Supplementary Data

130000 Supplementary Material
