# Supplementary material for: Active surveillance of acute paediatric hospitalisations demonstrates the impact of vaccination programmes and informs vaccine policy in Canada and Australia
Source: Euro Surveill. 2020 Jun 25;25(25):1900562. doi: 10.2807/1560-7917.ES.2020.25.25.1900562 (PMC7331140; doi:10.2807/1560-7917.ES.2020.25.25.1900562)
Supplement: Supplementary Material [file 1900562_TOP_Supplementary_Material.pdf]

This supplementary material is hosted by *Eurosurveillance* as supporting information alongside the article “Active surveillance of acute paediatric hospitalisations demonstrates the impact of vaccination programmes and informs vaccine policy in Canada and Australia”, on behalf of the authors, who remain responsible for the accuracy and appropriateness of the content. The same standards for ethics, copyright, attributions and permissions as for the article apply. Supplements are not edited by *Eurosurveillance* and the journal is not responsible for the maintenance of any links or email addresses provided therein.

SUPPLEMENTAL CONTENT 1

IMPACT and PAEDS contributions to surveillance of specific disease and syndromic targets

Acute flaccid paralysis (AFP)

IMPACT’s AFP surveillance contributed to Canada’s certification as polio-free in 1994 and continues to support Canada’s polio-free status. IMPACT centres contribute 50-70% of AFP cases captured through the Canadian Paediatric Surveillance Program, a network of over 2500 paediatricians across Canada.[1, 2] Despite IMPACT’s involvement, Canada continues to struggle to meet the WHO reporting target of 1 AFP case per 100,000 population.[1]

In Australia, AFP cases in children aged <15 years are reviewed by a polio expert panel.[3] As described in the main text, PAEDS reporting of AFP is key to Australia consistently meeting the WHO reporting target. In addition, AFP surveillance has facilitated data collection on specific conditions captured under this umbrella syndrome, such as Guillain Barré Syndrome (GBS), which enabled PAEDS to contribute to a multi-country study that identified a lack of association between GBS and pandemic H1N1 influenza vaccine in 2009-2010.[4]

Varicella

IMPACT monitored varicella and zoster-related hospitalizations from 1991 to 1996 (pre-vaccine era) and from 2000 to present, filling gaps in province-based surveillance.[5, 6] The

Canadian National Advisory Committee on Immunisation (NACI) used IMPACT data and estimates of the associated healthcare costs to inform single-dose varicella childhood immunization recommendations.[5, 7] Single dose varicella vaccination was implemented across jurisdictions between 2000 and 2006, with the second dose added between 2011 and 2016, using different vaccines [univalent and/or measles-mumps-rubella-varicella (MMRV)] at different ages (18 months or 4-6 years). IMPACT data from 2000-2015 showed that hospitalizations decreased by 66% from the pre-vaccine to 1-dose era and by 85% from the pre-vaccine to 2-dose era, with a 96% decrease in ICU days.[6, 8]

Varicella is not notifiable in all Australian jurisdictions and most notifications rely on laboratory diagnosis, which is known to be incomplete, especially in distinguishing varicella and zoster.[9] Active surveillance of children with varicella and herpes zoster commenced in 2007. Early data from PAEDS showed a 73% reduction in paediatric hospitalisations for varicella and 40% for herpes zoster following introduction of single-dose varicella vaccine to the National Immunisation Program (NIP) for 18-month olds.[10] Specimen genotyping over 12 years highlighted the contribution of the Oka vaccine strain compared to breakthrough (wild type) infection [3 of 126 hospitalized cases (2 immunocompromised, 1 zoster)].[11] Most hospitalised children (80%) were not vaccinated and a high proportion were immunocompromised (40%). However, vaccine effectiveness of Australia’s one dose varicella program against varicella hospitalisation estimated at 65% (95% CI: 43-78%) using PAEDS surveillance, remains suboptimal compared with the 2 dose program in Canada.[12] Varicella surveillance continues as an important PAEDS condition, since combination MMRV vaccine was introduced at 18 months of age in 2013 with a second dose for children recommended but not funded.[13]

Vaccine safety

In addition to identifying a new vaccine safety signal with BCG vaccine (see main text), IMPACT data has been used to demonstrate the ongoing safety of Canada's immunization programs.[14, 15] For example, a significant decrease in both hypotonic hyporesponsive episodes and seizures was observed following the switch from whole cell to acellular pertussis vaccine in Canada in 1996, providing evidence of the improved safety profile of acellular vaccines.[16] A review of encephalopathy cases with onset 0-7 days after pertussis immunization found no cases attributable to immunization following administration of ~6.5 million doses of acellular and whole-cell pertussis vaccines within IMPACT catchment areas.[17]

PAEDS activities with respect to monitoring of acute neurologic events, have been leveraged in two important ways. First, in the study of the incidence, age and geographic distribution, and aetiology of encephalitis, and second by broadening into evaluation of vaccine-proximate severe acute neurologic events (SANEs). All SANEs [captured via screening for encephalitis and AFP simultaneously, as well as severe febrile seizures (FS)] have been collected at different time periods, with those which are vaccine proximate (within 6 weeks) reported as AEFIs by PAEDS nurses to the Australian national Adverse Events Monitoring System (AEMS).

In addition, PAEDS studied FS following the introduction of MMRV vaccine to the NIP in 2013. This study documented the previously described short term 2-fold risk of FS post MMR vaccine (given at age 12 months) and absence of an increased risk of FS following monovalent varicella vaccine given at age 18 months.[18] Subsequently, introduction of MMRV vaccine at age 18 months, replacing monovalent varicella vaccine, was not associated with an increased risk of FS in a self-controlled case series analysis.[19]

Surveillance of intussusception through PAEDS rapidly identified a rotavirus vaccine-associated signal leading to an expansive national investigation.[20, 21] Due to the early and contemporaneous use of both rotavirus vaccines (Rotarix, GSK and RotaTeq, BioCSL/Merck and Co) in different jurisdictions in Australia, this surveillance provided comparative risk estimates for intussusception in the same context. PAEDS was the first published study of this risk in the world and has contributed to ongoing vaccine safety work regarding all new rotavirus vaccines since.[20-22] A study of the clinical severity of vaccine proximate and non-proximate intussusception nearing completion suggests no difference in intussusception outcomes post rotavirus vaccination.

References for Supplemental Content 1

[1] Pless R, Caron-Poulin L, Clow L. Acute Flaccid Paralysis. Canadian Paediatric Surveillance Program: 2017 Results. Ottawa, ON: Canadian Paediatric Surveillance Program; 2017. p. 10-1.
[2] Scheifele DW, Halperin SA, Gold R, Samson H, King A. Assuring vaccine safety: A celebration of 10 years of progress with the IMPACT project. Paediatr Child Health. 2002;7:645-8.
[3] Paterson BJ, Durrheim DN. Review of Australia's polio surveillance. Commun Dis Intell Q Rep. 2013;37:E149-55.
[4] Dodd CN, Romio SA, Black S, Vellozzi C, Andrews N, Sturkenboom M, et al. International collaboration to assess the risk of Guillain Barré syndrome following influenza A (H1N1) 2009 monovalent vaccines. Vaccine. 2013;31:4448-58.
[5] Law B, MacDonald N, Halperin S, Scheifele D, Dery P, Jadavji T, et al. The Immunization Monitoring Program Active (IMPACT) prospective five year study of Canadian children hospitalized for chickenpox or an associated complication. Pediatr Infect Dis J. 2000;19:1053-9.
[6] Tan B, McConnell A, Thibeault R, Jadavji T, Reyes Domingo F, Samson H, et al. Varicella hospitalizations in IMPACT (Immunization Monitoring Program, ACTive) centers after introduction of 1- and 2- dose varicella vaccination programs between 2000 and 2015. Canadian Paediatric Society annual meeting. Quebec City, QC2018.
[7] National Advisory Committee on Immunization. Statement on recommended use of varicella virus vaccine. Can Commun Dis Rep. 1999;25 (ACS-1):1-16.
[8] Tan B, Bettinger J, McConnell A, Scheifele D, Halperin S, Vaudry W, et al. The effect of funded varicella immunization programs on varicella-related hospitalizations in IMPACT centers, Canada, 2000-2008. Pediatr Infect Dis J. 2012;31:956-63.
[9] Sheel M, Beard F, Quinn H, Dey A, Kirk M, Koehler A, et al. Australian vaccine preventable disease epidemiological review series: varicella-zoster virus infections, 1998-2015. Commun Dis Intell (2018). 2018;42.

[10] Marshall HS, McIntyre P, Richmond P, Buttery JP, Royle JA, Gold MS, et al. Changes in patterns of hospitalized children with varicella and of associated varicella genotypes after introduction of varicella vaccine in Australia. Pediatr Infect Dis J. 2013;32:530-7.
[11] Marshall H, Clarke M, Heath C, Quinn H, Richmond P, Crawford N, Elliott E, Toi C, Kynaston A, Booy R, Macartney K, on behalf of the PAEDS investigators. Severe and complicated varicella and associated genotypes 10 years after introduction of a one dose varicella vaccine program. J Infect Dis. 2019;219:391-9.
[12] Quinn HE, Gidding HF, Marshall HS, Booy R, Elliott EJ, Richmond P, et al. Varicella vaccine effectiveness over 10 years in Australia; moderate protection from 1-dose program. J Infect. 2019;78:220-5.
[13] (ATAGI) ATAGoI. Australian Immunisation Handbook, Australian Government Department of Health. immunisationhandbook.health.gov.au.2 018.
[14] Scheifele D, Law B, Jadavji T. Disseminated bacille Calmette-Guerin infection: three recent Canadian cases. IMPACT. Immunization Monitoring Program, Active. Can Commun Dis Rep. 1998;24:69-72; discussion 73-5.
[15] Deeks SL, Clark M, Scheifele DW, Law BJ, Dawar M, Ahmadipour N, et al. Serious Adverse Events Associated with Bacille Calmette-Guerin Vaccine in Canada. Pediatr Infect Dis J. 2005;24:538-41.
[16] Le Saux N, Barrowman NJ, Moore DL, Whiting S, Scheifele D, Halperin S. Decrease in hospital admissions for febrile seizures and reports of hypotonic-hyporesponsive episodes presenting to hospital emergency departments since switching to acellular pertussis vaccine in Canada: a report from IMPACT. Pediatrics. 2003;112:e348.
[17] Moore DL, Le Saux N, Scheifele D, Halperin SA, Members of the Canadian Paediatric Society/Health Canada Immunization Monitoring Program A. Lack of evidence of encephalopathy related to pertussis vaccine: active surveillance by IMPACT, Canada, 1993-2002. Pediatr Infect Dis J. 2004;23:568-71.
[18] Macartney KK, Gidding HF, Trinh L, Wang H, McRae J, Crawford N, et al. Febrile seizures following measles and varicella vaccines in young children in Australia. Vaccine. 2015;33:1412-7.
[19] Macartney K, Gidding HF, Trinh L, Wang H, Dey A, Hull B, et al. Evaluation of Combination Measles-Mumps-Rubella-Varicella Vaccine Introduction in Australia. JAMA Pediatr. 2017;171:992-8.
[20] Buttery JP, Danchin MH, Lee KJ, Carlin JB, McIntyre PB, Elliott EJ, et al. Intussusception following rotavirus vaccine administration: post-marketing surveillance in the National Immunization Program in Australia. Vaccine. 2011;29:3061-6.
[21] Carlin JB, Macartney KK, Lee KJ, Quinn HE, Buttery J, Lopert R, et al. Intussusception risk and disease prevention associated with rotavirus vaccines in Australia's National Immunization Program. Clinic Infect Dis 2013;57:1427-34.
[22] Quinn HE, Wood NJ, Cannings KL, Dey A, Wang H, Menzies RI, et al. Intussusception after monovalent human rotavirus vaccine in Australia: severity and comparison of using healthcare database records versus case confirmation to assess risk. Pediatr Infect Dis J. 2014;33:959-65.

SUPPLEMENTAL CONTENT 2: Research approvals for IMPACT and PAEDS sites

IMPACT

Halifax, NS: IWK Health Centre Research Ethics Board (REB): #1002978 (Core surveillance), #1004728 (Rotavirus surveillance); Nova Scotia Health Authority REB ##1020676

Quebec City, QC: Comité d'éthique de la recherche du CHU de Québec-Université Laval : 47.05.02 (Core), 106.05.01 (Rotavirus)

Toronto, ON: The SickKids REB: #0019900593 (Core),#1000005043 (influenza), #1000034385 (Rotavirus)

Winnipeg, MB: Health Research Ethics Board: #HS15505 (core), #HS15508 (influenza), #HS10467 (rotavirus), #HS15506 (meningococcal)

Vancouver, BC: UBC C&W Research Ethics Board, H15-00782 (AEFI), H16-00515 (VPD), H16-00430 (VZV), H15-00954 (influenza), H17-01921 (RSV), H12-00366 (rotavirus), H04-70054 (meningococcal)

Ottawa, ON: CHEO REB, #10001163 (core), #10001106 (rotavirus)

Calgary, AB: Conjoint Health Research Ethics Board (CHREB), #REB15-1989_REN4 (AEFI), #REB14-1686_REN6 (H. influenzae), #REB17-1641_REN2 (RSV), #REB15-0943_REN5 (Meningococcal), #REB13-0405_REN6 (rotavirus)

Edmonton, AB: Health Research Ethics Board for University of Alberta, #Pro00000929 (core), #Pro00001735 (rotavirus), #Pro00000959_REN13 (meningococcal)

Montreal Children Hospital (Montreal, QC): Approved by the Director of Professional Services for surveillance

Sainte-Justine (Montreal): Comité d'éthique de la recherché du CHU Sainte-Justine, #1994-15, 108 (all targets)

St-John`s, NL: Health Research Ethics Board for Eastern Health, #1208.000 (all targets)

Saskatoon, SK: Biomedical Research Ethics Board for University of Saskatoon, #99-125 (all targets)

PAEDS

Sydney Children's Hospitals Network Human Research Ethics Committee HREC/18/SCHN/72 (covers 6 PAEDS sites)

Royal Darwin Hospital, NT: The Northern Territory Department of Health and Menzies School of Health Research, Approval number 2017 -2775
